# Study on TCM Syndrome Identification Modes of Coronary Heart Disease Based on Data Mining

**DOI:** 10.1155/2012/697028

**Published:** 2012-05-24

**Authors:** Qi Shi, Huihui Zhao, Jianxin Chen, Xueling Ma, Yi Yang, Chenglong Zheng, Wei Wang

**Affiliations:** Beijing University of Chinese Medicine, 11 Bei San Huan Dong Lu, ChaoYang District, Beijing 100029, China

## Abstract

Coronary heart disease (CHD) is one of the most important types of heart disease because of its high incidence and high mortality. TCM has played an important role in the treatment of CHD. Syndrome differentiation based on information from traditional four diagnostic methods has met challenges and questions with the rapid development and wide application of system biology. In this paper, methods of complex network and CHAID decision tree were applied to identify the TCM core syndromes of patients with CHD, and to establish TCM syndrome identification modes of CHD based on biological parameters. At the same time, external validation modes were also constructed to confirm the identification modes.

## 1. Introduction

Coronary heart disease (CHD) is one of the most important types of heart diseases because of its high incidence and high mortality. With the improvement of people's living standards, the prevalence tendency of CHD is rising and the population of youths suffering from CHD is growing. Coronary angiography has been considered as the “golden standard” in CHD diagnosis. CHD was called “thoracic obstruction” in TCM, with a variety of etiological factors; various clinical manifestations and complex syndromes [1]. Syndrome research has always been hot and difficult spots in TCM basic studies. Syndrome differentiation based on information from traditional four diagnostic methods has met challenges and questions with the rapid development and wide application of system biology. The “golden standard” of syndrome diagnosis has not been found yet. The large number and complexity, multilevel relationships of information from four diagnostic methods had constrained the accuracy of syndrome differentiation. Currently, the application of quantitative modes and data mining is developing rapidly [2, 3]. These technologies had provided approaches and methods for TCM syndrome differentiation. Our earlier study showed that the characteristics of information from four diagnostic methods above were in line with that of complex networks; not only in common with special nature on the basis of their own evolutionary mechanisms, but also closely contacted with nature and structural features. We also found that biological parameters could be considered as a reflection of the pathomechanism and physiological mechanism, which might be a reflection of syndrome in TCM too [4]. What is more, we have established a mode conducted by four biological parameters which could distinguish CHD patients with blood stasis syndrome from nonblood stasis syndrome patients by means of C5 Decision Tree [5]. This study indicated that core TCM syndromes could be identified by complex networks and biological parameters could be serviced as syndrome identification mode in CHD patients with the method of decision tree. 

## 2. Material and Methods

411 cases of CHD in-patients, aged from 45 to 75, from Anzhen Hospital (Beijing), hospitals of Traditional Chinese Medicine in Zhengzhou, Wuhan and Hubei Province, China-Japan Friendship Hospital (Beijing) and Dongzhimen Hospital (Beijing) (March 1, 2010 to June 30, 2011). All selected patients were diagnosed and confirmed by coronary angiography. Diagnosis standards of CHD refer to “Treatment Guide of Stable Angina” (ACC/AHA/ACP-ASIM, 1999) and “Diagnosis and Treatment Recommendations of Unstable Angina” (Chinese Society of Cardiology, 2000) [6, 7]. Diagnosis standards of TCM syndrome refer to “Guiding principles for the clinical study of Chinese Medicines” (2002) and “Terminology for Traditional Chinese Medicine clinical practice-Part of the syndrome” (1997) [8, 9]. All hospitalized patients had signed informed consent voluntarily. Excluded cases were patients who suffered from acute myocardial infarction, myocarditis, pericardial disease, cardiac neurosis, intercostal neuralgia, menopausal syndrome, or severe spondylosis; angina caused by rheumatic fever, syphilis, congenital coronary artery abnormalities, hypertrophic cardiomyopathy, aortic stenosis, or regurgitation; stroke, lung infection, nephritis, renal failure, urinary tract infections, rheumatism, severe arrhythmia, heart failure, cancer, other primary, and serious diseases of liver, kidney, hematopoietic system. Pregnant or lactating women, patients with allergies or psychosis, were also excluded. Demographic details of CHD patients with or without qi deficiency syndrome and phlegm-blood stasis syndrome were showed in Supplemental Tables 1 and 2 (See Supplementary material available online at doi: 10.1155/2012/697028).

### 2.1. TCM Syndrome Differentiation and Collections of Clinical Data

TCM syndrome was confirmed by three TCM deputy director physicians who had more than five years of clinical experiences. It should be performed within 24 hours since the patients were admitted to hospital. 90 clinical testing indicators from blood routine examination, urine routine examination, blood biochemical test, blood coagulation test, thyroid function, TNI, BNP, electrocardiogram (ECG), and echocardiography were collected within one week. 69 symptoms from four diagnostic methods were also collected, including chest pain, chest distress, short breath, cardiopalmus, cough, hypodynamia, spontaneous perspiration, night sweat, burning sensation of five centres, eyestrain, dry mouth, dizziness, amnesia, fainting feeling, tinnitus, insomnia, irritable tantrum, hypochondrium distending pain, sighing, depression, anorexia, abdominal distension, epigastric fullness, belching, nausea and vomiting, sore waist and knee, frequency of micturition at night, limb numbness, heel pain, pachylosis, obesity, white phlegm, yellow phlegm, frothy phlegm, tastelessness in the mouth, bitter taste in the mouth, sweet taste in the mouth, salty taste in the mouth, viscous and greasy taste in the mouth, yellow urine and oliguria, clear urine in large amounts, residual urine, cold abdomen and waist, heavy limbs, darkish complexion, red complexion, conjunctival congestion, dark color around eyes, dark red lip gingival, pale lips and finger nails, dark color in palatal mucosa, lower abdominal tenderness, faint low voice, emaciation, swollen tongue body, tooth-marked tongue, thick tongue coating, greasy tongue coating, thick and greasy tongue coating, yellow tongue coating, glossal petechia, lavender subglossal collateral vessels, blue purple subglossal collateral vessels, mauve subglossal collateral vessels, subglossal collateral vessels engorgement, deep pulse, thready pulse, uneven pulse, and weak pulse.

### 2.2. Data Processing of Four Diagnostic Information

We identified useful relationships among information from four diagnostic methods above by means of distance-based mutual information model (DMIM) [10]. Then, we established 120 association relationships among 69 symptoms from four diagnostic methods. The association data was consolidated into adjacency matrix and then converted into the format that Pajek software required.

### 2.3. Measurement of Network Properties and Complex Network Mapping

Pajek software 2.0 was used to analyze the node degrees and node core values of the four diagnostic information network. With the command of “Layout-Energy-Kamada-Kawai-Separate Components,” we drew the K-core network figures according to different colors and different degrees, mediated positions of the nodes with manual operation. Nodes and edges of the network could not be deleted. Then, we exported the network figures in Bitmap format.

### 2.4. Construction of Identification Modes and Validation

Data standardization was used to analyze information of the cases from different hospitals. Next, we establish two identification modes of CHD core syndromes by chi-square automatic interaction detection (CHAID) decision tree. “Qi deficiency” and “phlegm-blood stasis” were considered as dependent variable and 90 biological parameters were independent variables. We set “Parent Node” 50 and “Child Node” 25, allowing the tree model to grow sufficiently. 10-fold cross-validation was used in this research to minimize the bias produced by random sampling of the training and test data samples.

### 2.5. Construction of External Validation Modes

212 patients were selected from the 411 cases of CHD to establish new decision tree modes for external validation. Similarly, “qi deficiency” and “phlegm-blood stasis” were considered as dependent variable. 8 and 6 biological parameters got from research above were severed as independent variables. Due to the reduction in the number of independent variables, we set “Parent Node” 2 and “Child Node” 1 to allow the tree model growing much sufficiently. 10-fold cross validation was also used in this section for a validation.

## 3. Results

### 3.1. Results of Four Diagnostic Information Network Properties

Properties of four diagnostic information results showed that degree values of 69 nodes were from one to eleven. The degree values of subglossal collateral vessels engorgement, amnesia, faint low voice, white phlegm, heavy limbs, short breath, cough, anorexia, tastelessness in the mouth, as well as swollen tongue body were greater than six, and they indicated the core syndromes of CHD. The core deficiency syndrome was qi deficiency, and the core excessive syndrome was phlegm-blood stasis. From the results of network cores analysis, we found the core values of 31 four diagnostic information nodes were three. These nodes formed a 3-core network together (Table 1).

### 3.2. Color Classification Results of Four Diagnostic Information K-Core Network

**Figure 1:**
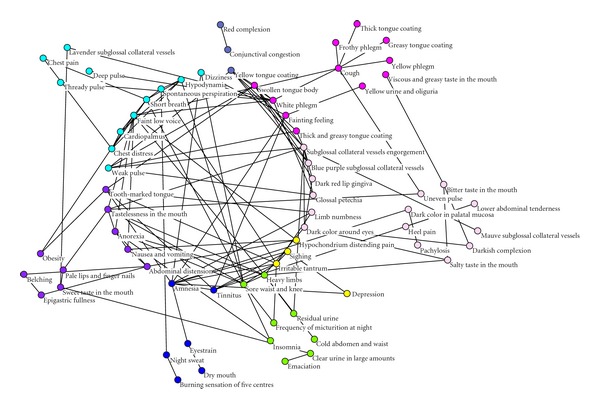
Color classification figure of information from four diagnostic methods K-Core network.

According to the core values of nodes, we drew the k-core network figure of information from four diagnostic methods (Figure 1). In the center of this network arrayed 31 nodes with the core value of 3, which were important for the network. Nodes with the same color indicated the same syndrome. 31 central nodes suggested 7 TCM syndromes: qi deficiency, qi stagnation, yin deficiency, yang deficiency, blood stasis, phlegm turbid, and heat syndrome. From the figure, qi deficiency, phlegm-blood stasis made up the basic syndromes of CHD patients. Dizziness, hypodynamia, spontaneous perspiration, short breath, faint low voice, cardiopalmus, chest distress, weak pulse, tooth-marked tongue, tastelessness in the mouth, anorexia formed qi deficiency syndrome; swollen tongue body, fainting feeling, white phlegm, as well as thick and greasy tongue coating were the performances of phlegm turbid syndrome. Blue purple subglossal collateral vessels, subglossal collateral vessels engorgement, glossal petechia, dark color around eyes, dark red lip gingival, as well as limb numbness were usually appeared in blood stasis patients.

### 3.3. Degree Classification Results of Four Diagnostic Information K-Core Network

**Figure 2:**
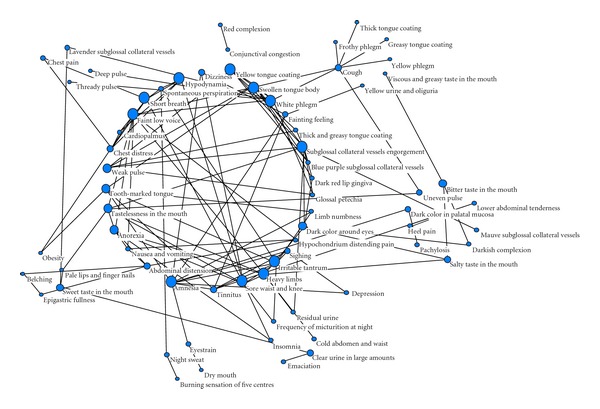
Degree Classification figure of information from four diagnostic methods K-Core network.

Figure 2 showed another expression form of four diagnostic information k-core network. In the photo, sizes of the circle represented the degree values of the nodes. Degree value was a simple but most important property of complex network. The degree value of one node was defined as the number of other nodes that connected to it. In a complex network, the greater degree value that one node was, the more significant role it had played. In qi deficiency syndrome, short breath, hypodynamia, faint low voice, weak pulse, as well as tastelessness in the mouth had the maximum degree values. Swollen tongue body, white phlegm, and cough had played major roles in phlegm turbid syndrome. Subglossal collateral vessels engorgement was the key nodes in blood stasis syndrome, whose degree value was eleven. In this network, we can determine the importance of the nodes by combinations of degree values and core values.

### 3.4. Results of Identification Mode for Qi Deficiency Syndrome

**Figure 3:**
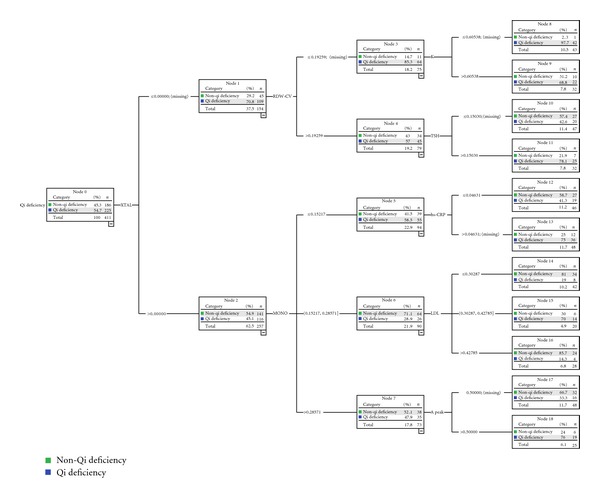
The 8 biological parameters made mode in identification Qi deficiency syndrome from 411 CHD patients.

Using the CHAID decision tree, an identification mode of qi deficiency was built with eight biological parameters. They were urine crystal (X TAL), erythrocyte distribution width-CV (RDW-CV), potassium ion (K), thyroid stimulating hormone (TSH), monocyte (MONO), high sensitive C-reactive protein (hs-CRP), low-density lipoprotein (LDL), and A peak in echocardiography. In this mode, there were 19 nodes and 11 terminal nodes and the tree depth was 3. From this mode we believed that the X TAL was the best predictive variable quantity of qi deficiency syndrome among the 8 parameters. However, we could not distinguish qi deficiency and non-qi deficiency completely only by X TAL. We could fall back on the second grade variable quantities: RDW-CV and MONO. The third grade variable quantities included K, TSH, hs-CRP, LDL, and A peak (Figure 3).

### 3.5. Results of Identification Mode for Phlegm-Blood Stasis Syndrome

**Figure 4:**
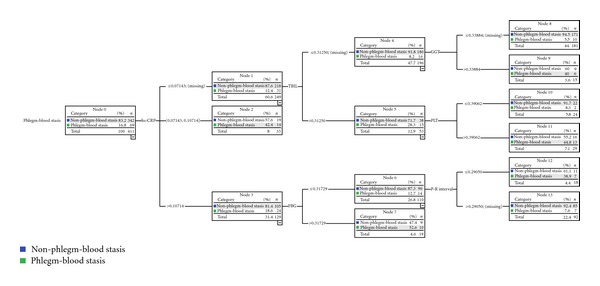
The 6 biological parameters made mode in identification phlegm-blood stasis syndrome from 411 CHD patients.

Identification mode of phlegm-blood stasis syndrome was made up of six properties: high-sensitive C-reactive protein (hs-CRP), total bilirubin (TBIL), glutamyltranspeptidase (GGT), platelet (PLT), fasting blood glucose (FBG), and P-R interval. The depth of this mode was 3. There were 14 nodes and 8 terminal nodes. The 6 parameters formed eight identification paths for phlegm-blood stasis syndrome. The best identification variable of the mode was hs-CRP. Hs-CRP was the only effective variable to identify phlegm-blood stasis syndrome if the value of hs-CRP was between 0.07143 and 0.10714. The second grade variable quantities were TBIL and FBG. The third grade variable quantities were GGT, PLT, and P-R interval (Figure 4).

### 3.6. Results of Validation for 411 Patients

The result of 10-fold cross-validation showed that in qi deficiency syndrome mode, 302 cases were predicted correctly, while the other 109 cases were wrong classified. The sensitivity and specificity of this mode were 70.2% and 77.4%. The percentage of correct prediction was 73.5%. In phlegm-blood stasis syndrome mode, 328 cases were predicted correctly, the other 83 cases were wrong classified. The sensitivity and specificity of this mode were 72.5% and 81.3%. The percentage of correct prediction was 79.8% (Table 2).

### 3.7. Results of External Validation Mode for Qi Deficiency Syndrome

**Figure 5:**
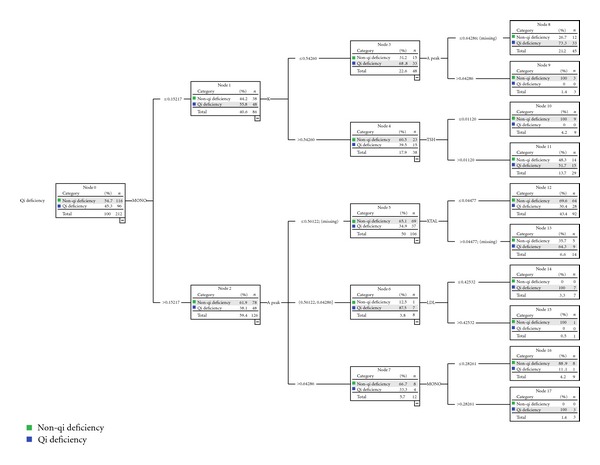
The 6 biological parameters made external validation mode in identification Qi deficiency syndrome from 211 CHD patients.

With the same CHAID decision tree method, an external validation mode of qi deficiency for 211 CHD patients was made up of six biological parameters. Unfortunately, this mode was lack of the parameters of hs-CRP and RDW-CV though we had made the tree model grow effectively as much as possible. The number of nodes in this mode was 18, and the number of terminal nodes was 10. MONO was the best predictive variable quantity of qi deficiency syndrome (Figure 5).

### 3.8. Results of External Validation Mode for Phlegm-Blood Stasis Syndrome

**Figure 6:**
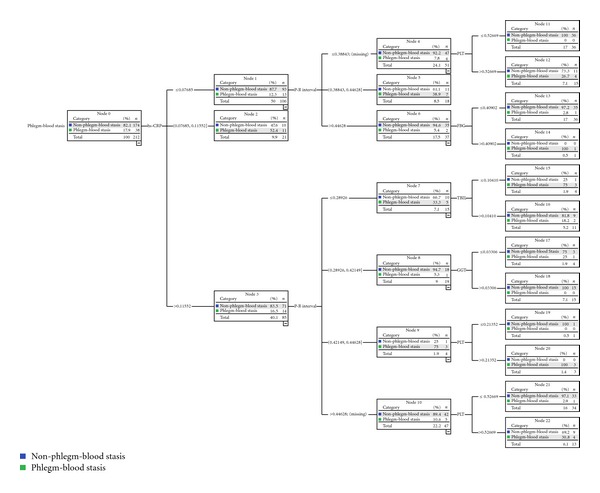
The 6 biological parameters made external validation mode in identification phlegm-blood stasis syndrome from 211 CHD patients.

External validation mode of phlegm-blood stasis syndrome included the same six properties compared with the identification mode above. There were 23 nodes and 14 terminal nodes in this mode. The mode was much more complex, for these 6 parameters formed 12 identification paths for phlegm-blood stasis syndrome. The best identification variable of the mode was still hs-CRP. The second grade variable quantity was P-R interval, and the third ones were the remaining four quantities (Figure 6).

### 3.9. Results of Validation for 212 Patients

The result of 10-fold cross-validation showed that in qi deficiency syndrome, external validation mode, the sensitivity and specificity were 69.8% and 73.3%. The percentage of correct prediction was 71.7%. In phlegm-blood stasis syndrome external validation mode, the sensitivity and specificity were 86.8% and 75.9%. The percentage of correct prediction was 77.8% (Table 3).

## 4. Discussion

Data mining is a method of extracting the database which is still unknown while useful information is implied potentially. It establishes a computer program, automatically scrutinizes in the database and tries to find modes or rules [11]. Complex networks can be used to describe the social relations among persons, kinships, network connections among computers, semantic relations among words, relations of cooperation between scientists, and so forth [12–14]. With the suggestion of small world network concept by Watts and Strogatz in 1998 [15], and with the development of pioneering study on scaling in random networks byBarabásiand Albert [16], more and more researchers had used complex networks in medical field. For example, researches on connection of the brain function [17], propagations of the diseases [18], studies of the drug efficacy and drug targets [19], gene regulatory networks [20], and interactions of protein [21].

The traditional approaches could not reveal the meaning of the four diagnostic information because the contents of them were numerous and the combination rules and relationships among the information were complex. TCM is a traditional medicine that capturing the variations of the disease based on the concept of wholism. Studies have shown that the diseases symptom networks had the characteristics of TCM syndromes classification [22]. In complex networks, the classification features, the demands of each role in the network organization and the relations of the elements in the progress of organization constitute are the potential force of the network [23]. The process of clinical diagnosis and treatment in TCM are also very similar to complex system. In the analysis of relationships among syndrome, therapeutic and Chinese herbal medicine, the main syndromes and monarch drugs were similar to the hubs of the network, the therapeutic methods and therapeutic principles were abstract summarization of the complex relations [24]. As one of the data mining methods, complex networks provided new methods and ideas for the studies of TCM. It explained the integrity, nonlinearity, and dynamic character of TCM from another point of view.

Pajek is a software that can analyze the data very fast and effective and a kind of simulation for complex network. Unlike the common network analysis software, Pajek can deal with the large-scale networks that contain millions of nodes and have broken the bottleneck that numbers of network analysis software can only process the small-scale data. It usually extracts small-scale networks from the large-scale ones in order to achieve a more detailed study by the classical algorithm and display the analysis results through powerful visualization capability [25, 26]. In many complex networks, there is a phenomenon that although the node number of the network is very large, but the “core” node number is still very small for the entire complex network. Intuitively, the “core” refers to the nodes that play important roles in the complex network. In a network, if any of the nodes has k neighbors that were also in this network, then the network is called “k-core network.” Researching the core of the complex network is to identify the entire “k-core network” in the complex network.

In our study, complex network was employed to identify the TCM core syndromes of CHD patients. The core syndromes included qi deficiency subjected to the deficiency syndromes, phlegm-blood stasis syndrome belonged to excessive syndromes. There are two reasons for the conclusions. Firstly, in this study, we gained a 3-core network, in the center of which arranged 31 nodes. These nodes played a major role. Among these nodes, there were 13 nodes reflected the qi deficiency syndrome, and 10 nodes reflected the phlegm-blood stasis syndrome. The 8 remaining nodes represented yin deficiency, yang deficiency, qi stagnation, and heat syndromes. Secondly, the degree value is a simple but most important property of complex network. The degree value of one node is defined as the number of other nodes that connected to it. In a complex network, the greater degree value that one node is, the more significant role it plays. In the network of four diagnostic information, the degree values of subglossal collateral vessels engorgement, amnesia, faint low voice, white phlegm, heavy limbs, short breath, cough, anorexia, tastelessness in the mouth, and swollen tongue body were higher than 6, most of which reflected the core syndromes we mentioned. The identifications of these core syndromes accurately laid the foundation for the constructions of syndrome identification modes by biological parameters.

Decision Tree is a decision support tool that uses a tree-like graph or model of decisions and their possible consequences, including chance event outcomes, resource costs, and utility. It is a way to display an algorithm. Decision trees are usually applied to cost-benefit studies, especially in decision-making analysis, to help identify a strategy most likely to reach a goal [27]. In many fields of clinical medicine, decision trees have been used successfully to solve complex and chaotic problems without mathematical models or a precise understanding of the mechanisms involved, such as genetic and molecular sequence analysis [28], hospital information system mining [29], and health care [30].

Chi-squared automatic interaction detector (CHAID) decision tree is a method of chi-square automatic interaction detection put forward by Kass in 1980 for the analysis of classification data [31]. It has the functions of target selection, variable selection, and clustering. Its core idea is to split the cases optimally according to the response variables and screened explanatory variables and to determine the grouping automatically of multiple contingency tables on the basis of significance results from chi-square test. The classification process of CHAID algorithm is described as follows. First, select the response variable of category, cross-classification goes into explanatory variables and response variables, then results in a series of two-dimensional classifications. Calculate the *χ*2 value of the two-dimensional classification, compare the *P* value. The best initial two-dimensional classification table with the minimum *P* value comes into being. Explanatory variables will continually be used to classify the response variables based on the best two-dimensional classification table. Repeat the process until the *P* value is greater than *α* value, then the classification stops and mode is formed [32]. Our previous results showed that CHAID decision tree can analyze the large and dormant data from clinical information due to the nonlinear relationship and the interactions between blood stasis syndrome and biological parameters.

The methods of syndrome studies cannot be completed without modern medicine. Due to the complexity itself, it is hard to find the “golden index” for syndrome identification. However, the combinations of different biological parameters may demonstrate the characteristics of different syndromes. Data mining methods have solved those problems mentioned above, which make it possible that macroinformation and microinformation could be combined effectively. Using the CHAID decision tree, an identification mode for qi deficiency syndrome was established with eight biological parameters, and another identification mode for phlegm-blood stasis syndrome was constructed with six biological parameters in our research. We could diagnose patients with or without qi deficiency by 11 paths and diagnose phlegm-blood stasis syndrome by 8 paths.

Studies showed that hs-CRP was significantly increased in CHD patients and had a moderate predictive value for CHD. It had a correlation with phlegm-blood stasis syndrome and provided objective basis for phlegm-blood syndrome differentiation [33]. Meanwhile, significant positive correlation was observed between hs-CRP and qi deficiency syndrome [34]. TBIL is a harmful metabolite in the body under the traditional view. In recent years, domestic and foreign researches showed that TBIL, as a kind of physiological oxidant, had played a role in antiarteriosclerosis. Low express of serum TBIL is an independent risk factor of CHD [35]. Serum GGT value may be the index of oxidative stress *in vivo*. The elevation of GGT can predict the myocardial infarction and stroke, and reflect the cell damage caused by oxygen free radical [36]. When the activated platelet adheres to the vessel wall, the platelet dusts (endothelial granules) are released. This process is closely related to the occurrence of CHD [37]. Study on the relationship between CHD and FBG proved CHD patients were more easily combined with abnormalities of FBG [38]. A correlation study on TCM syndromes and ventricular diastolic functions showed E peak decreased and A peak increased significantly in qi deficiency patients. It prompted the dysfunctions of heart early filling [39]. Some scholars believed that the elevation of RDW suggested the underlying inflammation of the body. Inflammation is one of the most important mechanisms of atherosclerosis. Increase of RDW may be a predictor of the CHD severity [40, 41]. The physiological functions of CHD patients with qi deficiency syndrome were weakened. When the promotion effect of qi was weakened, growth and development of the body would be hurt, physiological functions of the meridian and viscera declined for the earlier failure. Study showed in CHD patients with qi deficiency syndrome, the thyroxine (TH) decreased the ability to feedback regulate the pituitary. Correlations were found between TSH and CHD with qi deficiency syndrome [42]. Compared with healthy people, mononuclear cell count of CHD patients often increased. MONO may be the pathogenesis of CHD. Increases of MONO may indicate earlier happens of CHD especially in middle-aged people [43]. LDL is a reflection of the severity of coronary artery lesions. Its level increased with the aggravation and the severity of coronary lesions. Considering the prevention and treatment of CHD and the physiological need level of LDL, some scholars put forward the proper LDL level for 1.3–1.8 mmol/L [44].

In summary, it showed that application of CHAID decision tree may provide more biological indicator basis for TCM syndromes differentiation, which may also pave a way for further research on TCM syndrome.

## 5. Conclusion

Complex networks contributed a lot in the identification of the core TCM syndromes of CHD patients. We found that qi deficiency syndrome and phlegm-blood stasis syndrome were the basic syndromes of CHD patients in our study. Moreover, we established syndrome identification modes for CHD patients with or without core syndromes by CHAID decision tree. Qi deficiency identification mode included eight biological parameters: X TAL, RDW-CV, K, TSH, MONO, hs-CRP, LDL, and A peak. The accuracy of this mode was 73.5%, the sensitivity was 70.2% and specificity was 77.4%. The identification mode of phlegm-blood stasis syndrome included 6 biological parameters: hs-CRP, TBIL, GGT, PLT, FBG, and P-R interval, and the accuracy of this mode was 79.8%, the sensitivity was 72.5%, and the specificity was 81.3%. Constructions of the two external validation modes improved further reliabilities of the identification modes.

## Supplementary Material

Supplemental Table 1 and 2 showed the demographic details of CHD patients with or without qi deficiency syndrome and phlegm-blood stasis syndrome. There were no significant differences between the patients with or without qi deficiency syndrome and phlegm-blood stasis syndrome in the aspects of age, gender, BMI index, smoking or wine drinking situation, years of CHD, combination with arrhythmia, hypertension, diabetes, hypercholesterolemia, previous acute myocardial infarction, Previous cerebral infarction, and use of antiplatelet, anticoagulant, nitrate esters, statins, ACEI/ARB, beta blocker and calcium channel antagonist drugs.

## Figures and Tables

**Table 1:** Property values of four diagnostic information network.

Network nodes	Degree	Core value	Network nodes	Degree	Core value
chest pain	2	2	bitter taste in the mouth	3	2
chest distress	4	3	sweet taste in the mouth	3	2
short breath	7	3	salty taste in the mouth	3	2
cardiopalmus	3	3	viscous and greasy taste in the mouth	1	1
cough	7	2	yellow urine and oliguria	1	1
hypodynamia	5	3	clear urine in large amounts	2	1
spontaneous perspiration	4	3	residual urine	2	2
night sweat	2	1	cold abdomen and waist	1	1
burning sensation of five centres	1	1	heavy limbs	8	3
eyestrain	2	1	darkish complexion	2	2
dry mouth	1	1	red complexion	1	1
dizziness	3	3	conjunctival congestion	1	1
amnesia	10	3	dark color around eyes	5	3
fainting feeling	4	3	dark red lip gingiva	3	3
tinnitus	6	3	pale lips and finger nails	2	2
insomnia	2	2	dark color in palatal mucosa	2	1
irritable tantrum	8	3	lower abdominal tenderness"	2	1
hypochondrium distending pain	3	3	faint low voice	9	3
sighing	4	3	emaciation	1	1
depression	2	2	swollen tongue body	7	3
anorexia	7	3	tooth-marked tongue	6	3
abdominal distension	5	3	thick tongue coating	1	1
epigastric fullness	2	2	greasy tongue coating	1	1
belching	2	2	thick and greasy tongue coating	3	3
nausea and vomiting	4	3	yellow tongue coating	6	3
sore waist and knee	9	3	glossal petechia	3	3
frequency of micturition at night	2	2	lavender subglossal collateral vessels	2	2
limb numbness	3	3	blue purple subglossal collateral vessels	3	3
heel pain	1	1	mauve subglossal collateral vessels	1	1
pachylosis	1	1	subglossal collateral vessels engorgement	11	3
obesity	2	2	deep pulse	1	1
white phlegm	8	3	thready pulse	1	1
yellow phlegm	1	1	uneven pulse	2	2
frothy phlegm	1	1	weak pulse	5	3
tastelessness in the mouth	7	3			

**Table 2:** 10-fold cross-validation results of classification for 411 cases.

CHAID	TN	FP	Sensitivity (%)	Specificity (%)	Accuracy (%)
	FN	TP			
Qi deficiency	144	42	70.2%	77.4%	73.5%
	67	158			

Phlegm-blood stasis	278	64	72.5%	81.3%	79.8%
	19	50			

Note: sensitivity = TP/(TP + FN); specificity = TN/(TN + FP); accuracy = (TP + TN)/(TP + FP + TN + FN).

**Table 3:** 10-fold Cross-Validation results of classification for 212 cases.

CHAID	TN	FP	Sensitivity (%)	Specificity (%)	Accuracy (%)
	FN	TP			
Qi deficiency	85	31	69.8%	73.3%	71.7%
	29	67			

Phlegm-blood stasis	132	42	86.8%	75.9%	77.8%
	5	33			

Note: sensitivity = TP/(TP + FN); specificity = TN/(TN + FP); accuracy = (TP + TN)/(TP + FP + TN + FN).
