# Supplementary material for: Study on TCM Syndrome Identification Modes of Coronary Heart Disease Based on Data Mining
Source: Evid Based Complement Alternat Med. 2012 May 24;2012:697028. doi: 10.1155/2012/697028 (PMC3366225; doi:10.1155/2012/697028)
Supplement: Supplementary file 1 — Supplemental Table 1 and 2 showed the demographic details of CHD patients with or without qi deficiency syndrome and phlegm-blood stasis syndrome. There were no significant differences between the patients with or without qi deficiency syndrome and phlegm-blood stasis syndrome in the aspects of age, gender, BMI index, smoking or wine drinking situation, years of CHD, combination with arrhythmia, hypertension, diabetes, hypercholesterolemia, previous acute myocardial infarction, Previous cerebral infarction, and use of antiplatelet, anticoagulant, nitrate esters, statins, ACEI/ARB, beta blocker and calcium channel antagonist drugs. [file 697028.f1.docx]

The supplemental information is given below:

**Supplemental Table 1: Demographic Details of CHD Patients**

**with or without Qi Deficiency Syndrome**

|  | Qi Deficiency (n=224) | Non-Qi Deficiency (n=187) | *P* |
| --- | --- | --- | --- |
| Age | 62.20±8.74 | 59.39±8.42 | 0.870 |
| Gender(Male/Female) | 109/116 | 139/47 | 0.000 |
| Smoking | 59 (26.2%) | 75 (40.3%) | 0.000 |
| BMI | 24.68±4.10 | 26.25±3.72 | 0.000 |
| Wine Drinking | 34(15.1%) | 51(27.4%) | 0.002 |
| Years of CHD | 4.81±8.45 | 3.06±3.69 | 0.005 |
| [Arrhythmia](http://dict.cnki.net/dict_result.aspx?searchword=%e5%bf%83%e5%be%8b%e5%a4%b1%e5%b8%b8&tjType=sentence&style=&t=arrhythmia) | 24(10.7%) | 9(4.8%) | 0.030 |
| Hypertension | 161(71.6%) | 130(69.9%) | 0.712 |
| Diabetes | 82(36.4%) | 60(32.3%) | 0.374 |
| Hypercholesterolemia | 54(24.0%) | 39(21.0%) | 0.465 |
| Previous acute myocardial infarction | 15(6.7%) | 16(8.6%) | 0.460 |
| Previous cerebral infarction | 25(11.1%) | 7(3.8%) | 0.006 |
| Use of [antiplatelet drugs](http://dict.cnki.net/dict_result.aspx?searchword=%e6%8a%97%e8%a1%80%e5%b0%8f%e6%9d%bf%e8%8d%af%e7%89%a9&tjType=sentence&style=&t=antiplatelet+agents) | 195(86.7%) | 179(96.2%) | 0.001 |
| Use of [anticoagulant drugs](http://dict.cnki.net/dict_result.aspx?searchword=%e6%8a%97%e5%87%9d%e8%8d%af%e7%89%a9&tjType=sentence&style=&t=anticoagulant+drugs) | 5(2.2%) | 7(3.8%) | 0.356 |
| Use of nitrate esters drugs | 111(49.3%) | 101(54.3%) | 0.316 |
| Use of statins | 139(61.8%) | 123(66.1%) | 0.361 |
| Use of ACEI/ARB | 107(47.6%) | 87(46.8%) | 0.875 |
| Use of beta blocker | 127(56.4%) | 116(62.4%) | 0.224 |
| Use of calcium channel antagonist | 59(26.2%) | 44(23.7%) | 0.550 |

**Supplemental Table 2: Demographic Details of CHD Patients**

**with or without Phlegm-Blood Stasis Syndrome**

|  | Phlegm-Blood Stasis (n=69) | Non-Phlegm-Blood Stasis (n=342) | *P* |
| --- | --- | --- | --- |
| Age | 61.83±8.22 | 60.75±8.80 | 0.350 |
| Gender(Male/Female) | 46/23 | 202/140 | 0.239 |
| BMI | 26.92±4.90 | 25.08±3.74 | 0.005 |
| Smoking | 29(42.0%) | 105(30.7%) | 0.067 |
| Wine Drinking | 15(21.7%) | 70(20.5%) | 0.812 |
| Years of CHD | 3.65±4.41 | 4.09±7.16 | 0.624 |
| [Arrhythmia](http://dict.cnki.net/dict_result.aspx?searchword=%e5%bf%83%e5%be%8b%e5%a4%b1%e5%b8%b8&tjType=sentence&style=&t=arrhythmia) | 8(11.6%) | 25(7.3%) | 0.232 |
| Hypertension | 51(73.9%) | 240(70.2%) | 0.533 |
| Diabetes | 30(43.5%) | 112(32.7%) | 0.087 |
| Hypercholesterolemia | 21(30.4%) | 72(21.1%) | 0.089 |
| Previous acute myocardial infarction | 7(10.1%) | 24(7.0%) | 0.370 |
| Previous cerebral infarction | 6(8.7%) | 26(7.6%) | 0.757 |
| Use of [antiplatelet drugs](http://dict.cnki.net/dict_result.aspx?searchword=%e6%8a%97%e8%a1%80%e5%b0%8f%e6%9d%bf%e8%8d%af%e7%89%a9&tjType=sentence&style=&t=antiplatelet+agents) | 62(89.9%) | 312(91.2%) | 0.716 |
| Use of [anticoagulant drugs](http://dict.cnki.net/dict_result.aspx?searchword=%e6%8a%97%e5%87%9d%e8%8d%af%e7%89%a9&tjType=sentence&style=&t=anticoagulant+drugs) | 0(0%) | 12(3.5%) | - |
| Use of nitrate esters drugs | 37(53.6%) | 175(51.2%) | 0.710 |
| Use of statins | 44(63.8%) | 218(63.7%) | 0.997 |
| Use of ACEI/ARB | 36(52.2%) | 158(46.2%) | 0.364 |
| Use of beta blocker | 36(52.2%) | 207(60.5%) | 0.198 |
| Use of calcium channel antagonist | 18(26.1%) | 85(24.9%) | 0.829 |
